# Neural Stem Cells as a Novel Platform for Tumor-Specific Delivery of Therapeutic Antibodies

**DOI:** 10.1371/journal.pone.0008314

**Published:** 2009-12-15

**Authors:** Richard T. Frank, Marissa Edmiston, Stephen E. Kendall, Joseph Najbauer, Chia-Wei Cheung, Thewodros Kassa, Marianne Z. Metz, Seung U. Kim, Carlotta A. Glackin, Anna M. Wu, Paul J. Yazaki, Karen S. Aboody

**Affiliations:** 1 Divisions of Hematology and Hematopoietic Cell Transplantation, City of Hope National Medical Center and Beckman Research Institute, Duarte, California, United States of America; 2 Department of Molecular Medicine, City of Hope National Medical Center and Beckman Research Institute, Duarte, California, United States of America; 3 Department of Cancer Immunotherapy and Tumor Immunology, City of Hope National Medical Center and Beckman Research Institute, Duarte, California, United States of America; 4 Department of Neurosciences, City of Hope National Medical Center and Beckman Research Institute, Duarte, California, United States of America; 5 Department of Molecular and Medical Pharmacology, David Geffen School of Medicine, University of California Los Angeles, Los Angeles, California, United States of America; 6 Division of Neurology, Department of Medicine, UBC Hospital, University of British Columbia, Vancouver, British Columbia, Canada; 7 Institute for Regenerative Medicine, Gachon University Gil Hospital, Inchon, Korea; Roswell Park Cancer Institute, United States of America

## Abstract

**Background:**

Recombinant monoclonal antibodies have emerged as important tools for cancer therapy. Despite the promise shown by antibody-based therapies, the large molecular size of antibodies limits their ability to efficiently penetrate solid tumors and precludes efficient crossing of the blood-brain-barrier into the central nervous system (CNS). Consequently, poorly vascularized solid tumors and CNS metastases cannot be effectively treated by intravenously-injected antibodies. The inherent tumor-tropic properties of human neural stem cells (NSCs) can potentially be harnessed to overcome these obstacles and significantly improve cancer immunotherapy. Intravenously-delivered NSCs preferentially migrate to primary and metastatic tumor sites within and outside the CNS. Therefore, we hypothesized that NSCs could serve as an ideal cellular delivery platform for targeting antibodies to malignant tumors.

**Methods and Findings:**

As proof-of-concept, we selected Herceptin™ (trastuzumab), a monoclonal antibody widely used to treat HER2-overexpressing breast cancer. HER2 overexpression in breast cancer is highly correlated with CNS metastases, which are inaccessible to trastuzumab therapy. Therefore, NSC-mediated delivery of trastuzumab may improve its therapeutic efficacy. Here we report, for the first time, that human NSCs can be genetically modified to secrete anti-HER2 immunoglobulin molecules. These NSC-secreted antibodies assemble properly, possess tumor cell-binding affinity and specificity, and can effectively inhibit the proliferation of HER2-overexpressing breast cancer cells *in vitro*. We also demonstrate that immunoglobulin-secreting NSCs exhibit preferential tropism to tumor cells *in vivo*, and can deliver antibodies to human breast cancer xenografts in mice.

**Conclusions:**

Taken together, these results suggest that NSCs modified to secrete HER2-targeting antibodies constitute a promising novel platform for targeted cancer immunotherapy. Specifically, this NSC-mediated antibody delivery system has the potential to significantly improve clinical outcome for patients with HER2-overexpressing breast cancer.

## Introduction

Recombinant monoclonal antibodies offer a targeted approach to cancer therapy and have substantially lower toxicity to normal cells than chemotherapy. However, their large molecular size and high affinity to antigens at the tumor border limit the ability of antibodies to deeply penetrate tumor foci [1]. Central nervous system (CNS) tumors pose a particular challenge for antibody therapeutics, because the blood-brain barrier (BBB) prevents intravenously-injected antibodies from reaching CNS metastases.

The unique tumor-tropic properties of neural stem cells (NSCs) may provide the means to significantly improve antibody distribution within metastatic tumor sites throughout the body [2], [3], [4], [5], [6]. NSCs localize to infiltrative tumor cells and hypoxic tumor regions, which are inaccessible to intravenously administered drugs [7]. NSCs are also able to cross the BBB, making them a potential platform for therapeutic antibody delivery to the brain. Previous studies have demonstrated that HB1.F3 cells, a clonal, well-characterized human NSC line [8], can effectively express and deliver a variety of therapeutic agents to primary and metastatic tumor sites, including metastases to the brain [2], [4], [5], [6], [9]. Therefore, we hypothesized that NSCs could be used to enhance antibody-based therapies by delivering antibodies to previously inaccessible tumor foci, while minimizing the exposure of normal tissue to the therapeutic agent.

As a model system, we chose Herceptin™ (trastuzumab), a well characterized antibody therapy for HER2-overexpressing breast carcinoma, because HER2-overexpression is correlated with a poor prognosis and an increased incidence of CNS metastases [10]. Trastuzumab is a humanized monoclonal antibody that targets the HER2 receptor, which is overexpressed in approximately 20–30% of human breast carcinomas [11]. Although the clinical use of trastuzumab has made measurable improvements in the prognosis of patients with HER2-overexpressing breast cancer, its effectiveness is limited [12], [13]. In addition, HER2 is expressed in many normal tissues, giving systemic HER2-targeted antibody therapy the potential to cause toxicity in a subset of patients, including cardiac myopathy, congestive heart failure, and pulmonary toxicity [14], [15].

Here we report that HB1.F3 NSCs can be engineered to express and secrete functional full-length HER2-specific human immunoglobulin molecules that can selectively bind to and inhibit the proliferation of HER2-positive breast carcinoma cells. Furthermore, these antibody-secreting NSCs retain tropism to tumor cells *in vitro* and can deliver anti-HER2 antibody to tumor foci *in vivo*. Our data suggest that NSCs may represent a novel platform for delivery of therapeutic antibodies to metastatic breast carcinoma.

## Results

### NSCs Can Express and Secrete Anti-HER2 Antibody

**Figure 1:**
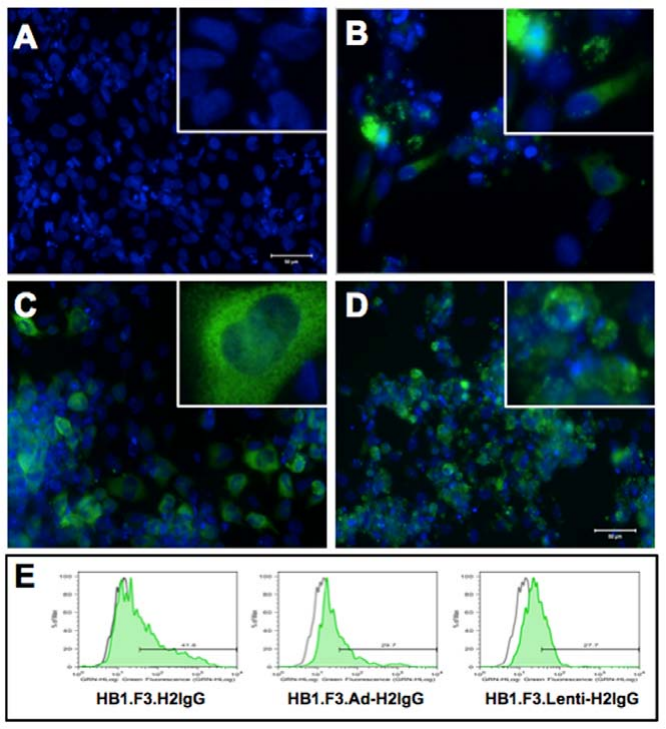
Expression of human IgG in NSCs. Fluorescence micrograph of parental HB1.F3 NSCs (**A**), HB1.F3.H2IgG (**B**), HB1.F3.Adeno-H2IgG NSCs (**C**), and HB1.F3.Lenti-H2IgG (**D**). Cells were stained with anti-human IgG (green) and DAPI nuclear stain (blue). Expression was confirmed by intracellular flow cytometry of fixed and permeabilized NSCs (**E**). Fluorescence of fixed and permeabilized parental NSCs (indicated by gray histogram) was used to set the marker for each graph.

To determine whether NSCs can express immunoglobulins, HB1.F3 NSCs were transiently co-transfected with plasmids encoding the heavy and light chains of an anti-HER2 antibody (HB1.F3.H2IgG) identical in sequence to trastuzumab. Intracellular expression of human immunoglobulin in HB1.F3.H2IgG NSCs was visualized using immunocytochemistry. In contrast to parental HB1.F3 NSCs (Fig. 1A), HB1.F3.H2IgG NSCs displayed a clear intracellular signal when stained with FITC-conjugated anti-human IgG (Fig. 1B). This signal was often punctate and localized to the cytoplasm, which is consistent with expected cellular localization within the endoplasmic reticulum and secretory vesicles. We next explored viral expression of anti-HER2 antibody in NSCs in order to prolong antibody expression. Trastuzumab heavy and light chains were reformatted into a bicistronic expression cassette and cloned into adenoviral and lentiviral vectors for transient and stable expression, respectively. Expression of human immunoglobulin within adenovirally-transduced HB1.F3 NSCs (HB1.F3.Ad-H2IgG; Fig. 1C) and HB1.F3.Lenti-H2IgG, a stable cell line generated by lentiviral transduction of HB1.F3 NSCs (Fig. 1D), was visualized using immunocytochemistry. These results were confirmed by intracellular flow cytometry (Fig. 1E). Both transient transfection with Lipofectamine and transduction with adenovirus resulted in a subpopulation of cells that experessed human IgG, as shown by the increased fluorescence intensity. In contrast, the stable HB1.F3.Lenti-H2IgG cell line showed a shift in green fluorescence intensity for the entire cell population, albeit at lower levels than with the transient expression methods. This is consistent with a stable cell line.

**Figure 2:**
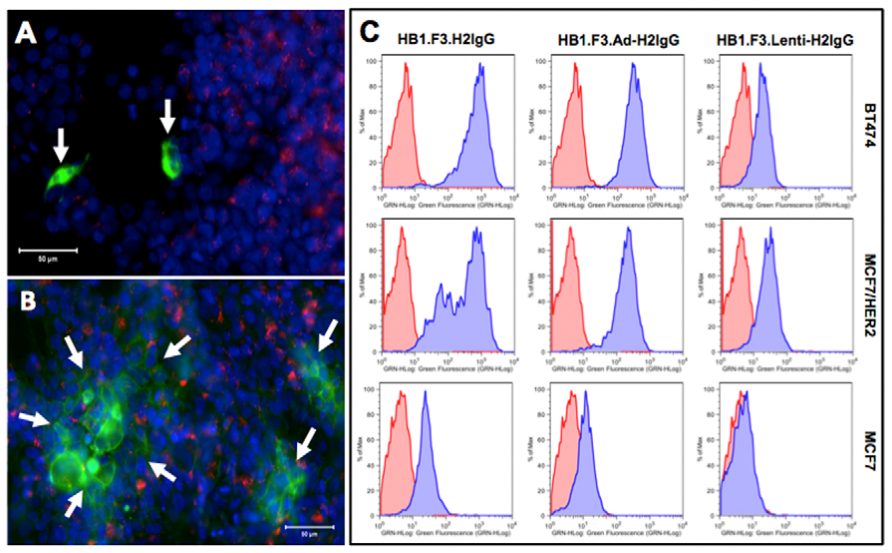
NSC-secreted human IgG specifically binds HER2. Transfected NSCs were co-cultured with CM-DiI-labeled (red) MCF7 (**A**) or MCF7/HER2 cells (**B**) and stained with FITC-conjugated anti-human IgG (green) and DAPI (blue). Arrows indicate NSCs expressing human IgG that does not bind to adjacent MCF7 control cells (**A**) or areas of NSC-secreted human IgG bound to MCF7/HER2 target cells (**B**). Bar, 50 µm. Flow cytometric analysis of BT474, MCF7/HER2, or MCF7 target cells after incubation with supernatant from HB1.F3.H2IgG, HB1.F3.Adeno-H2IgG, or HB1.F3.Lenti-H2IgG (**C**), followed by incubation with FITC-conjugated anti-human IgG (blue histograms). Red histograms on each graph show target cells incubated with supernatant from unmodified HB1.F3 NSCs as a negative control.

To determine whether the NSC-secreted anti-HER2 antibody binds specifically to the HER2 receptor, HB1.F3-H2IgG cells were co-cultured overnight with either MCF7 human breast cancer cells that express low levels of HER2, or MCF7/HER2 cells, an MCF7 line stably transfected to overexpress HER2 [16]. Breast cancer cells were pre-labeled with CM-DiI to visually distinguish them from NSCs in co-culture. MCF7/HER2 cells in the vicinity of transfected NSCs were strongly labeled with human IgG (Fig. 2B). In contrast, we did not detect any human immunoglobulin bound to the surface of neighboring MCF7 cells (Fig. 2A). To confirm specificity, supernatant from transfected or transduced HB1.F3 cells was incubated with MCF7, MCF7/HER2, or BT474, a human breast cancer cell line that endogenously overexpresses HER2. Antibody bound to breast cancer cells was detected by flow cytometry (Fig. 2C), and significant quantities of human IgG were detected on the surface of both HER2-overexpressing cell lines following incubation with supernatant from HB1.F3-H2IgG and HB1.F3.Ad-H2IgG NSCs. Supernatant from stable HB1.F3.Lenti-H2IgG NSCs also labeled HER2-overexpressing breast cancer cells, but to a lesser extent. This result is consistent with the lower expression levels observed by intracellular flow cytometry of HB1.F3.Lenti-H2IgG cells. Importantly, each supernatant labeled MCF7 cells between one and two logs less than either of the HER2-overexpressing breast cancer cell lines. These findings demonstrate the NSC-secreted antibody is specific for HER2.

### NSC-Secreted IgG Is Functionally Equivalent to Trastuzumab

To directly compare trastuzumab and NSC-secreted anti-HER2 antibody (F3-IgG), F3-IgG was purified from the supernatant of transfected HB1.F3 cells. Trastuzumab and F3-IgG were analyzed by SDS-PAGE under reducing and non-reducing conditions. Both antibodies showed an apparent molecular mass of 150 kDa under non-reducing conditions and two major bands indicative of the heavy (50 kDa) and light (25 kDa) chains under reducing conditions (data not shown). This suggests the two antibodies have a similar molecular composition.

**Figure 3:**
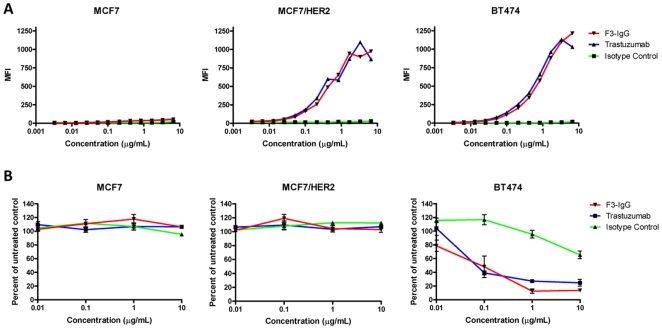
NSC-secreted anti-HER2 antibody is functionally equivalent to trastuzumab. Flow cytometric analysis (**A**) of MCF7, MCF7/HER2, and BT474 cells labeled with F3-IgG, trastuzumab, or a human IgG isotype control antibody. Graphs show mean fluorescence intensity (MFI) of labeled cells. Inhibition of cell proliferation (**B**) of MCF7, MCF7/HER2, or BT474 cells treated for 6 days with F3-IgG, trastuzumab, or isotype control antibody. Graphs show proliferation as a percentage of untreated cells.

To confirm the specificity of F3-IgG, purified F3-IgG was added directly to target cells and binding was detected by flow cytometry. At all concentrations tested, trastuzumab and purified F3-IgG bound equally well to MCF7/HER2 and BT474, as indicated by similar mean fluorescence intensities of assayed target cells (Fig. 3A). A non-specific isotype control antibody (human IgG1) showed no reactivity. Minimal labeling of MCF7 cells was observed for both trastuzumab and F3-IgG. This indicates that F3-IgG binds to HER2 protein specifically and with an affinity comparable to that of trastuzumab, which suggests that it should have similar anti-tumor efficacy.

To assess the ability of F3-IgG to inhibit the proliferation of HER2-overexpressing breast carcinoma cells, we measured the proliferation of BT474, MCF7/HER2 and MCF7 breast cancer cell lines in the presence of trastuzumab, F3-IgG, or an isotype-matched control antibody. F3-IgG significantly inhibited the proliferation of BT474 cells in a dose-dependent manner, comparable to the trastuzumab (Fig. 3B). As expected, MCF7 cells were not affected by treatment with either trastuzumab or F3-IgG. F3-IgG did not inhibit the proliferation of MCF7/HER2 cells, which is consistent with previously published reports for trastuzumab [16], [17].

### Antibody-Secreting NSCs Display Tumor Tropism and Deliver Antibody to Breast Cancer Xenografts

**Figure 4:**
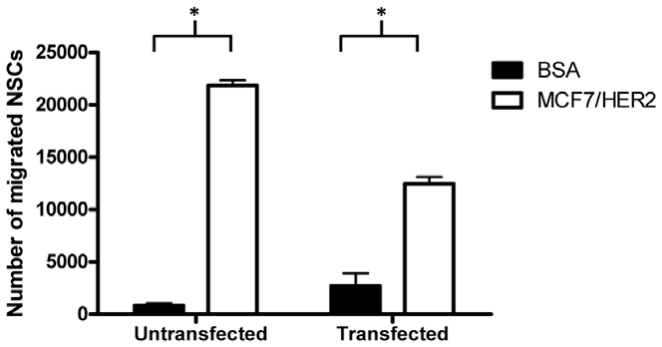
*In vitro* migration of NSCs to breast carcinoma conditioned media. Migration of parental NSCs and anti-HER2-transfected HB1.F3 NSCs to breast tumor-conditioned media in an *in vitro* chemotaxis assay. In this assay, bovine serum albumin (BSA) was used as a negative control for chemotaxis. Both parental and transfected NSC lines preferentially migrated to MCF7/HER2 compared to negative control (2% BSA) (*p*<0.01).

In order for stem cell targeting of antibodies to be clinically relevant, immunoglobulin-secreting NSCs must retain their migratory tumor-tropic properties. We tested the migration of transfected NSCs *in vitro* using a chemotaxis assay in which cells must actively migrate through a semi-permeable membrane in response to a cytokine gradient. Both parental HB1.F3 NSCs and NSCs expressing intact anti-HER2 immunoglobulin showed preferential *in vitro* migration to tumor cell-conditioned media. Although we observed fewer migrated anti-HER2-expressing NSCs than untransfected NSCs, both cell types showed a statistically significant tropism to MCF7/HER2 conditioned medium relative to BSA control (Fig. 4). This result indicates that immunoglobulin-expressing HB1.F3 NSCs would likely maintain the *in vivo* tumor tropism of the parental NSC line.

**Figure 5:**
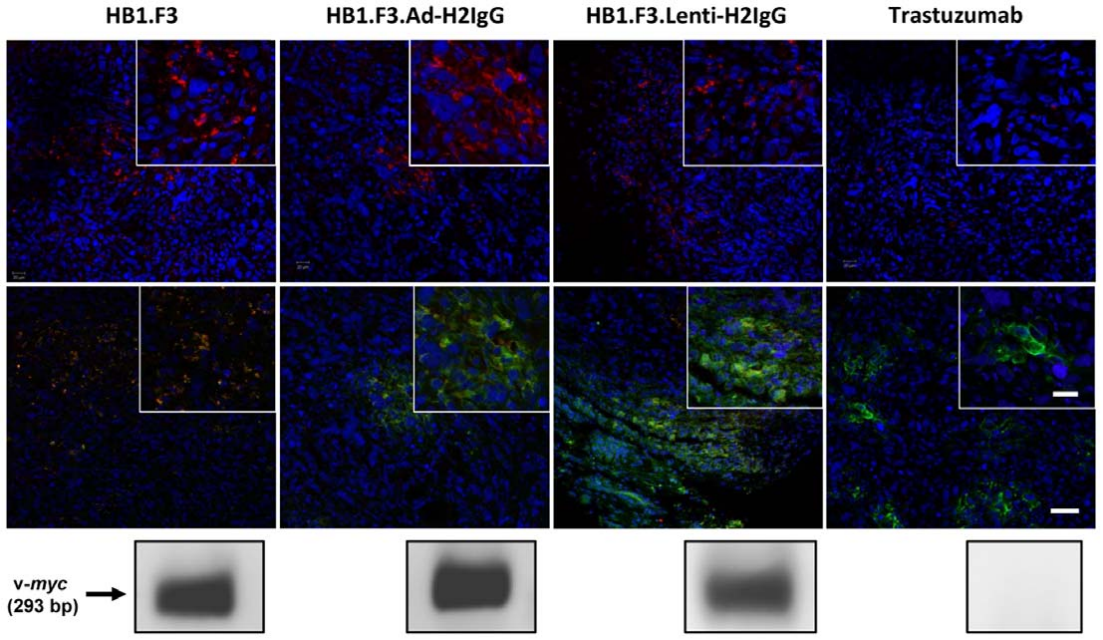
NSCs target breast carcinoma and can deliver anti-HER2 antibody *in vivo*. Confocal fluorescence micrographs of tumor sections from MCF7/HER2 xenografts. First three panels in the upper row show the presence of CM-DiI-labeled red NSCs (HB1.F3, HB1.F3.Ad-H2IgG, HB1.F3.Lenti-H2IgG, respectively) in tumors 4 days after intravenous injection. The fourth panel of the upper row shows tumor with no red NSCs in mice treated with trastuzumab alone (sporadic small red dots not associated with cells are visible as autofluorescence background). Middle row shows tumor sections stained with FITC-conjugated anti-human IgG (green). Bar, 50 µm. Insets are 2× magnification (Bar, 20 µm). Bottom row shows confirmation of the presence or absence of HB1.F3 NSCs within tumors by PCR detection of a DNA amplicon (293 bp) of the v*-myc* transgene, a unique identifier of the HB1.F3 cell line.

We next tested the ability of antibody-expressing NSCs to deliver anti-HER2 antibodies to tumor foci *in vivo* using a xenograft nude-beige mouse model. Intravenously-injected parental and transduced HB1.F3 NSCs were detected within the tumor mass of each treated animal by immunohistochemistry. Tumor sections from animals receiving NSCs showed a patchy distribution of NSCs (CM-DiI labeled, red) within the tumor. In contrast, tumor sections from mice receiving trastuzumab injections showed no red stained cells (Fig. 5). The presence of NSCs within the tumor mass was confirmed by detection of v*-myc* DNA using nested PCR. A 293 bp PCR product was detected in the tumors of every mouse treated with HB1.F3, HB1.F3.Ad-H2IgG, or HB1.F3.Lenti-H2IgG. In contrast, no PCR product was detected in tumors from mice treated with trastuzumab alone. Tumor sections were then stained with FITC-conjugated anti-human IgG. Tumor sections from trastuzumab-injected animals showed patches of bright green cobblestone patterns, indicative of antibody bound to tumor cell membranes. As expected, trastuzumab distribution was heterogeneous and localized in the vicinity of tumor vasculature. Tumor sections from mice treated with HB1.F3.Ad-H2IgG and HB1.F3.Lenti-H2IgG also showed patches of green cobblestone patterns throughout the tumor mass. Antibody-expressing NSC appear yellow, due to the presence of both CM-DiI and FITC-conjugated anti-human IgG. Parental HB1.F3 NSCs showed only background levels of green fluorescence and tumor sections from mice injected with these NSCs did not exhibit the green cobblestone pattern associated with membrane-bound antibody.

Of note, quantitative ELISA revealed significant quantities of human IgG in the blood of trastuzumab-injected mice. In contrast, mice treated with antibody-expressing NSCs showed anti-HER2 IgG at the tumor site, but no human IgG was detectable in the blood (data not shown). Taken together, these data indicate that anti-HER2 IgG-expressing NSCs can deliver antibody selectively to tumor foci *in vivo*.

## Discussion

Overexpression of HER2 is a marker for poor prognosis in breast cancer and is often correlated with increased metastasis, including to the CNS [18], [19]. Trastuzumab directly inhibits the proliferation of HER2-overexpressing breast cancer cells and induces immune-mediated killing of target cancer cells through antibody-dependent cellular cytotoxicity [20]. However, trastuzumab is unable to cross the BBB, and therefore cannot be used to treat CNS metastases. Importantly, NSCs can target breast cancer metastasis in the brain [9] and can penetrate deep and hypoxic regions of solid tumors [7]. Therefore, we examined the feasibility of expressing therapeutic antibodies in tumor-tropic human NSCs, with the ultimate goal of using such NSCs as a platform for delivery of anti-HER2 antibody to otherwise inaccessible tumor foci.

NSCs have been successfully engineered to express and deliver a broad range of recombinant therapeutic proteins to tumor foci [3]. However, antibodies are complex tetrameric molecules, that are normally expressed only by lymphocytes and a few other specialized cells. Whether NSCs are capable of expressing and secreting such complex molecules was previously unknown. Here, we have demonstrated that NSCs can express and secrete intact immunoglobulin while retaining their inherent tumor tropism. In addition, F3-IgG binds to HER2 specifically and inhibits the proliferation of HER2-overexpressing breast cancer cells.

In our initial experiments, we transiently co-transfected trastuzumab heavy and light chains with Lipofectamine to achieve trastuzumab expression in HB1.F3 NSCs. Although this method was successful in generating high levels of human IgG in HB1.F3 NSCs, expression was limited to 3–4 days and caused unpredictable toxicity to the NSCs. We therefore chose to pursue two alternative viral transduction strategies to prolong antibody expression. Our laboratory has previously used adenoviral expression as a means to transiently express therapeutic transgenes [2], [6]. We have found that adenoviral transduction results in high levels of transgene expression for at least two weeks. We have also generated a stable anti-HER2 IgG-expressing NSC line (HB1.F3.Lenti-H2IgG). In this stable line, antibody expression is prolonged, but at significantly lower levels than observed with adenovirally-transduced NSCs (HB1.F3.Ad-H2IgG). Both of these strategies resulted in antibody-expressing NSCs capable of achieving tumor-specific antibody delivery *in vivo*. Our ability to detect anti-HER2 antibody at the tumor site but not in the blood of NSC-treated mice suggests that this approach could have robust localized anti-tumor effect, while minimizing the systemic toxicity associated with traditional trastuzumab therapy.

The use of NSCs to target antibodies specifically to tumor foci has broad implications for the field of antibody therapy in cancer and other diseases. In addition to improving distribution of antibodies to the CNS and within malignant solid tumors, this strategy could expand the number of targets to which antibodies can be directed. Currently, molecular targets for cancer therapy must either be exclusively expressed by tumor cells or expressed at significantly higher levels in tumors than in normal tissues. Because the tumor specificity of NSC-mediated immunotherapy is endowed by the NSCs, antigens on tumor cells can likely be safely targeted, regardless of whether they are present in healthy tissues.

We conclude that tumor-tropic human NSCs can be genetically modified to secrete intact therapeutic antibodies. The data presented here provide the first fundamental step in the development of a stem cell-based cancer immunotherapy. Such a therapeutic platform has significant clinical implications for improving the treatment of metastatic breast carcinoma and other malignant solid tumors.

## Materials and Methods

### Vector Construction and Purification

DNA encoding the trastuzumab scFv molecule (hu4D5v8) was generated as described previously [21]. The genes encoding the variable domains were individually inserted on to the human IgG1 heavy and light chains by splice-overlap PCR using the anti-CEA hT84.66-M5A antibody as a template and cloned into glutamine synthetase vectors (Lonza Biologics, Slough, UK) as previously described [21]. To create viral expression vectors, a bicistronic construct was created. Briefly, trastuzumab heavy and light chain sequences were amplified with PCR and a truncated FMDV 2A peptide sequence was inserted between them by splice overlap extension. This expression construct was inserted into the pENTR vector of Invitrogen's Gateway system using the D-TOPO cloning kit. Entry vector constructs were recombined into adenoviral (pAd, Invitrogen) or lentiviral (pCSC-Zeo with Gateway recombination sites cloned into the multiple cloning site) destination vectors according to manufacturer's instructions. Adenovirus was produced by transfecting adenoviral expression vector into E1-complemented cell line 293A and performing successive rounds of amplification until high titer was achieved. Virus was then purified using ViraBind Adenovirus Purification Kit (Cell Biolabs). Lentivirus was produced by cotransfecting replication incompetent lentiviral vector with psPAX2 (Addgene, 12260) and pLP/VSV-G (Invitrogen). Virus was purified from crude viral supernatant using LentiX Viral concentrator (Clontech) according to manufacturer's instructions.

### Cell Culture

HB1.F3, MCF7, MCF7/HER2, 293A and 293FT cells were cultured in Dulbecco's Modified Eagle Medium (DMEM) supplemented with 10% fetal bovine serum (FBS), 100 units penicillin, 100 µg/mL streptomycin, and 2 mM L-glutamine. BT474 cells were cultured in RPMI 1640 supplemented with 10% FBS, 100 units penicillin, 100 µg/mL streptomycin, and 2 mM L-glutamine and 500 µg/mL insulin. All cells were cultured at 37°C with 6% CO_2_.

### Transfection and Transduction of HB1.F3 NSCs

Plasmids encoding the trastuzumab heavy and light chains, pEE6-HER2-Heavy and pEE12-HER2-Light, were co-transfected in equal concentrations using Lipofectamine LTX (Invitrogen) according to the manufacturer's instructions. Dishes (10 cm diameter) were seeded with HB1.F3 NSCs (3×10^6^) and transfected with pooled heavy and light chain DNA (6 µg). Stable trastuzumab-expressing NSCs (HB1.F3.Lenti-H2IgG) were generated by incubating HB1.F3 cells with purified lentivirus (MOI 100) for 4 h in the presence of 8 µg/mL polybrene. After 24 h, trastuzumab-expressing NSC were selected using increasing concentrations of Zeocin (InvivoGen), beginning at 100 µg/mL. For adenoviral transduction, HB1.F3 cells were incubated with purified adenovirus (1∶1000 dilution in DMEM) for 4 h and then cultured overnight in DMEM.

### Mouse Tumor Xenograft Model

Female 7-week old nude-beige-xid mice (NCI) were injected with MCF7/HER2 cells (5×10^6^) in the right mammary fat pad. Tumors were allowed to grow until palpable (3 weeks), then mice were injected intravenously with trastuzumab (4 mg/kg) or unmodified NSCs (2×10^6^), adenovirally-transduced NSCs or HB1.F3/H2IgG (3 mice per group). One additional mouse per group received an intratumoral injection as a control. Four days later, mice were humanely euthanized and tumors were harvested and fixed for 48 h in 4% PFA. Blood was collected by cardiac puncture immediately after euthanasia. All procedures were approved by City of Hope IACUC.

### PCR Detection of v*-myc*

Genomic DNA from tumor sections was purified using DNeasy Blood and Tissue kit (Qiagen). Nested PCR was performed as described previously [2]. The v*-myc* amplicon was detected as a band of 293 bp. Genomic DNA from HB1.F3.H2IgG cells was used as a positive control.

### Quantitative ELISA

Mouse serum was diluted 100 or 1000-fold in PBS and tested by quantitative ELISA using the human IgG ELISA Kit (Bethyl Laboratories) according to manufacturer's instructions.

### Immunocytochemistry and Immunohistochemistry

Parental or transfected/transduced NSCs were seeded into 4-well chamber slides and allowed to adhere overnight. For co-culture experiments, CM-DiI-labeled breast cancer cells were seeded one day prior to the addition of NSCs. Adherent cells were washed once (PBS supplemented with 100 mg/L calcium chloride and 100 mg/L magnesium chloride), fixed (4% paraformaldehyde, 10 min), then permeabilized (0.3% Triton X-100 in PBS, 30 min). For tissue sections, PFA-fixed tumors were impregnated with 30% sucrose then cut into 10 µm sections using a cryostat. Sections were blocked and stained overnight with FITC-conjugated donkey anti-human IgG (H+L) (Jackson ImmunoResearch). Slides were washed, counterstained with 4′,6-diamidino-2-phenylindole (DAPI), mounted in fluorescent mounting media (DAKO), and imaged using a Nikon Eclipse TE2000-U microscope equipped with a SPOT RT Slider digital camera or confocal imaging using a Zeiss LSM 510 confocal microscope (Carl Zeiss Microimaging).

### Purification of Secreted Antibody

Immunoglobulin-containing HB1.F3.H2IgG NSC culture supernatant was subjected to purification by protein A affinity chromatography. The NSC-expressed anti-HER2 antibody and trastuzumab were analyzed by SDS-PAGE as previously described [21].

### Flow Cytometry

The specificity of purified NSC-secreted antibody (F3-IgG) was compared to trastuzumab by measuring the binding to HER2 over-expressing target cells. Bound immunoglobulin on target cells was labeled with FITC-conjugated donkey anti-human IgG (H+L) (Jackson ImmunoResearch) and detected using a Guava EasyCyte flow cytometer. For intracellular flow cytometry, cells were fixed in 4% PFA for 20 min, then washed and resuspended in permeabilization buffer (PBS with 3% FBS and 0.1% Saponin) prior to staining.

### Cell Proliferation Assay

Breast carcinoma cells (5×10^3^ cells/well) were seeded into a 96-well plate and treated with trastuzumab, isotype-matched control IgG, or F3-IgG at concentrations ranging from 0.01 to 10 µg/mL. As a negative control, medium alone (100 µL) was added. Plates were incubated (37°C) for 5 or 6 days. To quantify cell proliferation, WST-1 reagent (Roche) was used according to manufacturer's instructions. Background absorbance of WST-1 with media only was subtracted and all concentrations were expressed as a percentage of control. All experiments were performed in triplicate.

### *In Vitro* Chemotaxis Assay

Cell migration assay was performed as described previously [22]. Briefly, NSCs (1×10^5^ in 400 µL culture media) were seeded into the upper chamber of a 24-well Boyden chamber with an 8 µm pore-size membrane, and tumor cell-conditioned medium (600 µL) was added to the bottom chamber. After 4 h, the number of migrated cells was quantified using a Guava EasyCyte flow cytometer. NSCs transfected with anti-HER2 antibody were used 24 h after transfection, the time-point that correlated with peak antibody expression. All experiments were performed in triplicate. Student's *t*-test was used for statistical analysis. *p*<0.05 was considered significant.
